# Expanded prevalence of group 4 capsule expression and its genetic requirements in a broad array of *Shigella* strains

**DOI:** 10.1128/msphere.00354-26

**Published:** 2026-08-12

**Authors:** Brock W. Brethour, Tao Wu, Bing Guo, Megan D. Broussard, David A. Rasko, Eileen M. Barry

**Affiliations:** 1 Center for Vaccine Development and Global Health, University of Maryland School of Medicine 12264, Baltimore, Maryland, USA; 2 Department of Microbiology and Immunology, University of Maryland School of Medicine 12264, Baltimore, Maryland, USA; 3 Institute for Genome Sciences, University of Maryland School of Medicine 12264, Baltimore, Maryland, USA; 4 Department of Microbial Pathogenesis, University of Maryland School of Medicine 12264, Baltimore, Maryland, USA; Vanderbilt University Medical Center, Nashville, Tennessee, USA

**Keywords:** *Shigella*, host–pathogen interactions, capsule, group 4 capsule, O-antigen

## Abstract

The expression of a group 4 capsule (G4C) has been identified in *Shigella sonnei* and *Shigella flexneri* serotype 6 and demonstrated to play a significant role in pathogenic processes in *S. sonnei*. However, it has not been examined phenotypically in *S. flexneri* 6. Despite the impact of G4C, no systematic analysis of its expression or the presence of the genomic determinants has been performed across a large collection of *S. sonnei* and *S. flexneri* serotypes 2a and 6 or any of the other >46 *Shigella* strains and serotypes that cause disease. Our examination of the genomes of 1,219 geographically diverse *Shigella* isolates revealed the presence of intact *gfc* operons not only in the majority (≥98%) of *S. sonnei* and *S. flexneri* 6 strains but also, surprisingly, in the majority (≥97%) of *Shigella boydii* and *Shigella dysenteriae* strains. Approximately 95% of non-serotype 6 *S. flexneri* isolates contained a range of diverse *gfc* mutations preventing G4C expression; the remaining 5% contained an intact *gfc* operon yet had single-nucleotide polymorphisms within the promoter that resulted in lack of expression. These findings establish that no *S. flexneri* serotypes other than 6 express a G4C. Capsule expression was directly linked to reduced epithelial cell invasion for all strains examined, confirming differences in pathogenic processes between capsule-positive and capsule-negative *Shigella* strains. This work identifies a more widespread expression of capsule among *Shigella* strains than previously appreciated and identifies critical genetic requirements.

**IMPORTANCE:**

*Shigella* is the leading cause of moderate-to-severe diarrhea in 1- to 5-year-old children in low-resource settings, yet there is no licensed vaccine. Despite the impact of the group 4 capsule (G4C) on *Shigella* epithelial cell invasion, its expression has not been determined for the majority of *Shigella* species and serotypes that cause disease. This study investigated the presence of intact genes necessary for G4C expression in 1,219 geographically diverse *Shigella* isolates and examined phenotypes associated with G4C expression. We report that G4C is much more widespread than previously appreciated; it is expressed in the vast majority of *Shigella sonnei* and *Shigella flexneri* 6 clinical isolates, as well as *Shigella boydii* and *Shigella dysenteriae* strains. No *S. flexneri* serotypes outside of serotype 6 expressed a G4C. This study provides evidence that G4C expression in *Shigella* is much more widespread than previously recognized and emphasizes the importance of capsule expression in host–pathogen interactions.

## INTRODUCTION

The Global Enteric Multicenter Study (GEMS) identified *Shigella* among the top 4 causes of moderate-to-severe diarrhea (MSD) in children 1 year of age and under and the number one pathogen of 1- to 5-year-old children in sites throughout sub-Saharan Africa and South Asia (1, 2). The subsequent Vaccine Impact of Diarrhea in Africa (VIDA) study in sub-Saharan Africa from 2015 to 2018 determined that *Shigella* had the greatest attributable incidence of any pathogen for MSD in children 1–5 years of age (3). *Shigella* are Gram-negative bacteria that cause MSD in multiple populations, including children in low-resource settings and travelers, such as military personnel (4–7). There are four species within the *Shigella* genus, including *Shigella sonnei* (*Ss*), *Shigella flexneri* (*Sf*), *Shigella boydii* (*Sb*), and *Shigella dysenteriae* (*Sd*) (4, 8). In the GEMS and VIDA studies, *Sf* was the most prevalent *Shigella* species identified, 65% and 68% respectively, while *Ss* was the second most common, 23% and 18% respectively (3, 9). *Sb* and *Sd* combined was reported as 10.4% of cases in GEMS and 14.1% of cases in VIDA (1, 3).

*Sf* has more than 15 serotypes, determined by the O-antigen (O-Ag), which are recovered at various rates in clinical and surveillance samples (4, 10–12). The GEMS and VIDA studies identified *Sf* serotypes 2a, 3a, and 6 as the three most commonly isolated serotypes from children with MSD, followed by *Sf*1b, 2b, and 4a. Protective immunity is believed to be O-Ag specific, and vaccine strategies have targeted the most prevalent O-Ag serotypes (13–15). While epidemiological studies invariably identify *Sf* as the most prevalent species in low- to middle-income countries, *Ss* is the most common *Shigella* species identified in high-resource settings and increases in incidence in countries as they develop economically (16–19). *Ss* is more genetically conserved compared to *Sf* and contains a single serotype (4, 20). There has been a rapid emergence of extensively drug-resistant *Ss*, highlighting the urgent need to include *Ss* together with *Sf* in an efficacious multivalent *Shigella* vaccine (21, 22).

While serotype classification remains relevant for *Shigella* identification as well as O-Ag antibody protection, recent studies have defined phylogroups of *Shigella* based on genomic content (8, 23). These comparative genomic analyses, as well as mechanistic studies, reveal key differences among and within the *Shigella* species and serotypes, concluding that virulence-associated processes can differ between different *Shigella* strains (23–28). The seminal studies defining virulence mechanisms and pathogenesis of *Shigella* primarily used *Sf*2a 2457T and *Sf*5a M90T as the model strains (29). Genomic differences reported between *Shigella* species and serotypes underscore the need for more in-depth examination of functional virulence mechanisms among a larger array of strains and serotypes beyond the limited archetype isolates.

One important virulence factor identified on *Ss* is the presence of a group 4 capsule (G4C) (30, 31). G4Cs are unique in that the polysaccharide composition is the same as that of the LPS O-Ag (32, 33). O-Ag biosynthesis and export is a Wzx/Wzy-dependent process; in *Ss* exclusively, the O-Ag biosynthesis genes are located on the virulence plasmid (pINV), whereas the export genes are encoded on the chromosome (34–36). The O-Ag subunits are polymerized by Wzy in the periplasm, where the modal length is determined by the chain length determinant WzzB (37–39). A portion of the O-Ag chains are ligated to the lipid-A-core by the WaaL ligase, forming the completed LPS molecule (40). Alternatively, O-Ag chains can be exported to the surface via the products of the *gfc* operon to form a capsule that is attached to an unknown anchor (31, 41). The *gfc* operon consists of seven genes: *gfcABCDE*, *etp*, and *etk*. G4Cs have been identified in enteropathogenic *Escherichia coli and* enterohemorrhagic *E. coli* (EHEC), where all seven genes were demonstrated to be required for capsule expression (31).

Expression of a G4C in *Ss* has been shown to reduce invasion in HeLa cells, decrease inflammation in rabbit ileal loops, and decrease complement-mediated lysis (30, 35). Additionally, the presence of a capsule in *Sd* serotype 1 has previously been reported (42, 43). Expression of G4C has also been reported in *Sf* serotype 6 confirmed by biochemical methods, and the *gfc operon* genes have been reported to be present in *Sd* (31, 44, 45), but it is unknown if they are intact and functional. A 14-bp deletion in *etk* in *Sf*2a strains 2457T and 301 has been reported to prevent G4C expression in these strains (30).

Despite the significant role of the capsule in the pathogenic process, there has been no systematic examination of *Shigella* isolates for this feature. In this study, we examined a large collection of geographically diverse *Shigella* clinical isolates from the GEMS study and detected partial, mutated, or intact *gfc* operons. We identified *Sf* isolates from multiple serotypes containing *gfc* operons with intact open reading frames that lacked expression. We evaluated functional outcomes associated with G4C expression. A more complete understanding of G4C expression and its impact on pathogenesis among different *Shigella* species and serotypes will support the development of novel therapeutic interventions and efficacious vaccines for *Shigella*.

## RESULTS

### Comparisons of *gfc* operon sequences show diverse genetic integrity both between and within serotypes

The presence of the *gfc* operon has been noted for several *Shigella* species and strains, including *Ss* 53G, *Sd* M131649, *Sf2*a 2457T, and 301 and *Sf*6 CCH060 (30, 31, 45). Early studies reported the presence of intact operons in all of the above strains, while later studies identified a 14-bp deletion in *etk* in *Sf*2a 2457T, which eliminated G4C expression (30). To better understand the distribution of *gfc* operon sequences within *Shigella*, we performed a large-scale genetic analysis of 1,219 *Shigella* isolates from the GEMS. We identified an intact operon in over 99% (304/305) of the *Ss* strains examined, as well as 98% (140/143) of the *Sf*6 strains analyzed (Table 1). Surprisingly, we observed that 97% (58/60) of *Sd* and 100% (71/71) of *Sb* genomes examined included an intact *gfc* operon (Table 1).

**TABLE 1:** Species distribution of intact *gfc* operon

Species	Total no. of genomes	Complete *gfc* operons present	No. of isolates with mutation(s) resulting in non-functional *gfc* operon	% with intact *gfc* operon^*d*^
*S. dysenteriae*	60	58	2^*a*^	97
*S. boydii*	71	71	0	100
*S. sonnei*	305	304	1^*b*^	99.7
*S. flexneri* serotype 6	143	140	3^*c*^	98

^*a*^ Two strains had the *gfc* gene cluster distributed on three contigs; thus, it was impossible to determine if it was intact.

^*b*^ One isolate did not contain the *gfc* operon.

^*c*^ Three different strains contained either an SNP that resulted in a stop codon in *etk*, a 5-bp deletion, or was distributed on three different contigs; thus, it was impossible to determine if it was intact.

^*d*^ Intact is defined as no insertions or deletions present, as well as no SNPs that result in a stop codon.

**Fig 1:**
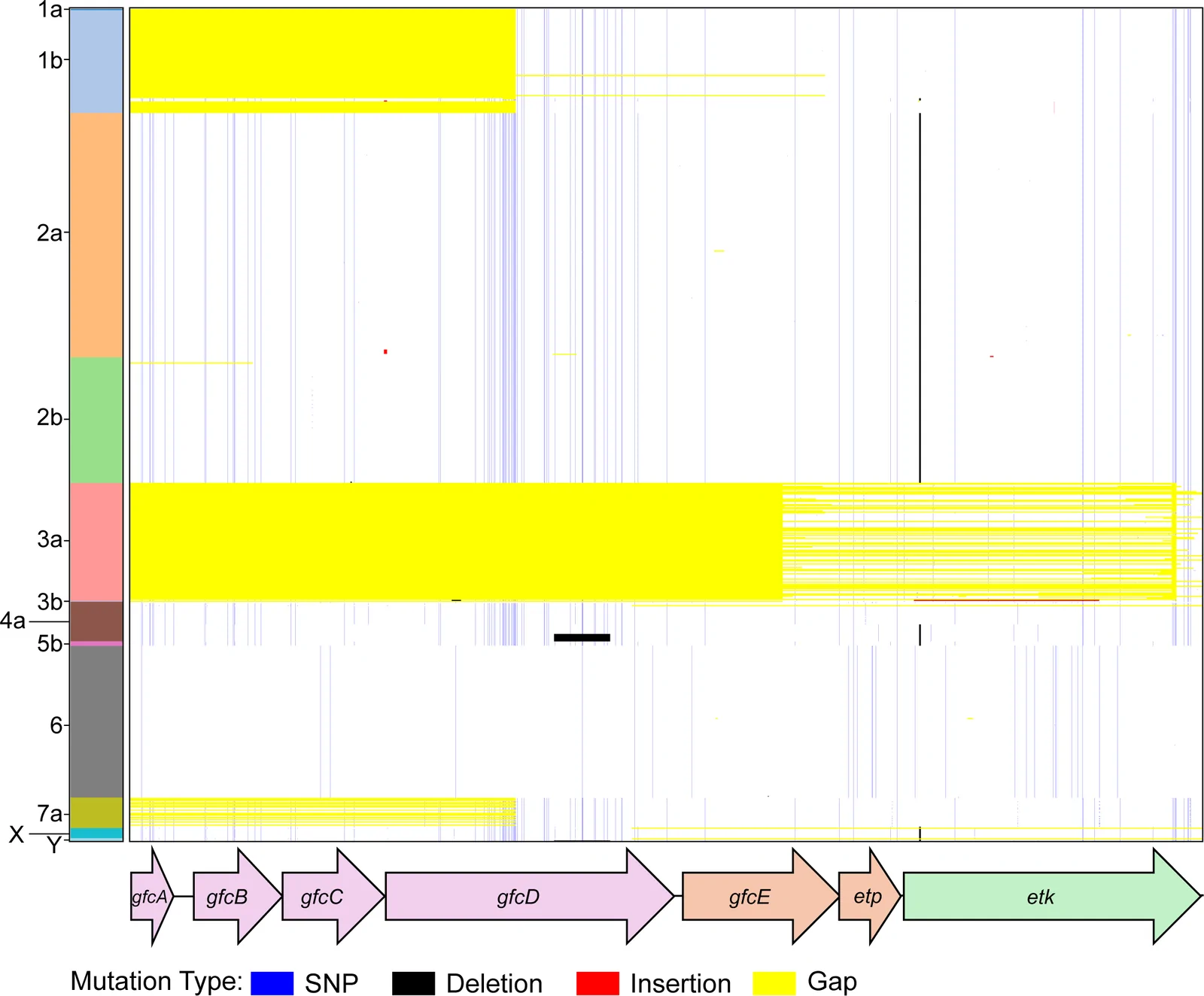
Genetic determination of the *gfc* operon within *Sf*. Schematic diagram of mutations of 640 different *Sf* strains broken down by serotype (*Y*-axis) compared to reference strain *Ss*046 along the *gfc* operon (*X*-axis). Blue indicates a SNP; red indicates insertion; black indicates deletion; and yellow indicates a gap compared to *Ss*046.

Our analysis also determined that the 14-bp deletion in *etk* previously reported in *Sf*2a 2457T was present in 100% (229/229) of the *Sf*2a strains analyzed (Table 2). The sequence of the *gfc* operon was highly conserved within geographically diverse *Sf*2a (99.98% identity) as well as within *Sf*6 strains (99.99% identity). G4C genotypes and phenotypes of other *Sf* serotypes have not been previously described. Our analysis identified the same 14-bp *etk* deletion in *etk* in 100% of *Sf*2b and *Sf*5b isolates examined. Fifty-one percent of *Sf* strains, including a variety of serotypes, lacked the 14-bp deletion (Table 2). An examination across the entire *gfc* operon revealed that there was a range of mutation patterns outside of the 14-bp *etk* deletion. Isolates from other serotypes, including some *Sf*1b and *Sf*7a, were missing the first third of the *gfc* operon, *gfcABCD*, whereas *Sf*3a isolates were missing the majority of the operon with a number containing fragments of *gfcE*, *etp*, and *etk* across multiple contigs (Fig. 1). There were multiple single-nucleotide polymorphisms (SNPs) identified in *gfc* genes of *Sf* isolates compared to *Ss*, yet only one instance of a SNP resulting in a stop codon was observed among all strains examined, in *Sf*6 602660. Most mutations that resulted in expected loss of function were insertions or deletions. *Sf*6 has a distinct *gfc* SNP pattern compared to other *Sf* serotypes, in alignment with the previously described reports regarding the genomic diversity of *Sf*6 compared to other *Sf* (Fig. 1) (46, 47). Other *Sf* serotypes outside of serotype 6, including *Sf*4a and *Sf*7a, contained isolates with SNPs compared to *Ss*, but none with expected loss of protein translation and function, which should allow capsule production (Table 2). Fifty-four percent (20/37) of *Sf*4a isolates contained an intact *gfc* operon, while 39% (11/28) of *Sf*7a isolates contained a genetically intact *gfc* operon.

**TABLE 2:** *S. flexneri* serotype distribution of an intact *gfc* operon

Serotype	Total no. of genomes	14-bp *etk* deletion	Complete *gfc* operons present	No. of isolates with mutation(s) resulting in a non-functional *gfc* operon	% with intact *gfc* operon
1a	2	0	0	2	0
1b	97	2	0	97	0
2a	229	229	0	229	0
2b	118	118	0	118	0
3a	111	0	0	111	0
3b	1	0	0	1	0
4a	37	16	20	17	54
5b	4	4	0	4	0
6	143	0	140	3	98
7a	28	1	11	17	39
Y	3	2	0	3	0
X	10	9	0	10	0

**Fig 2:**
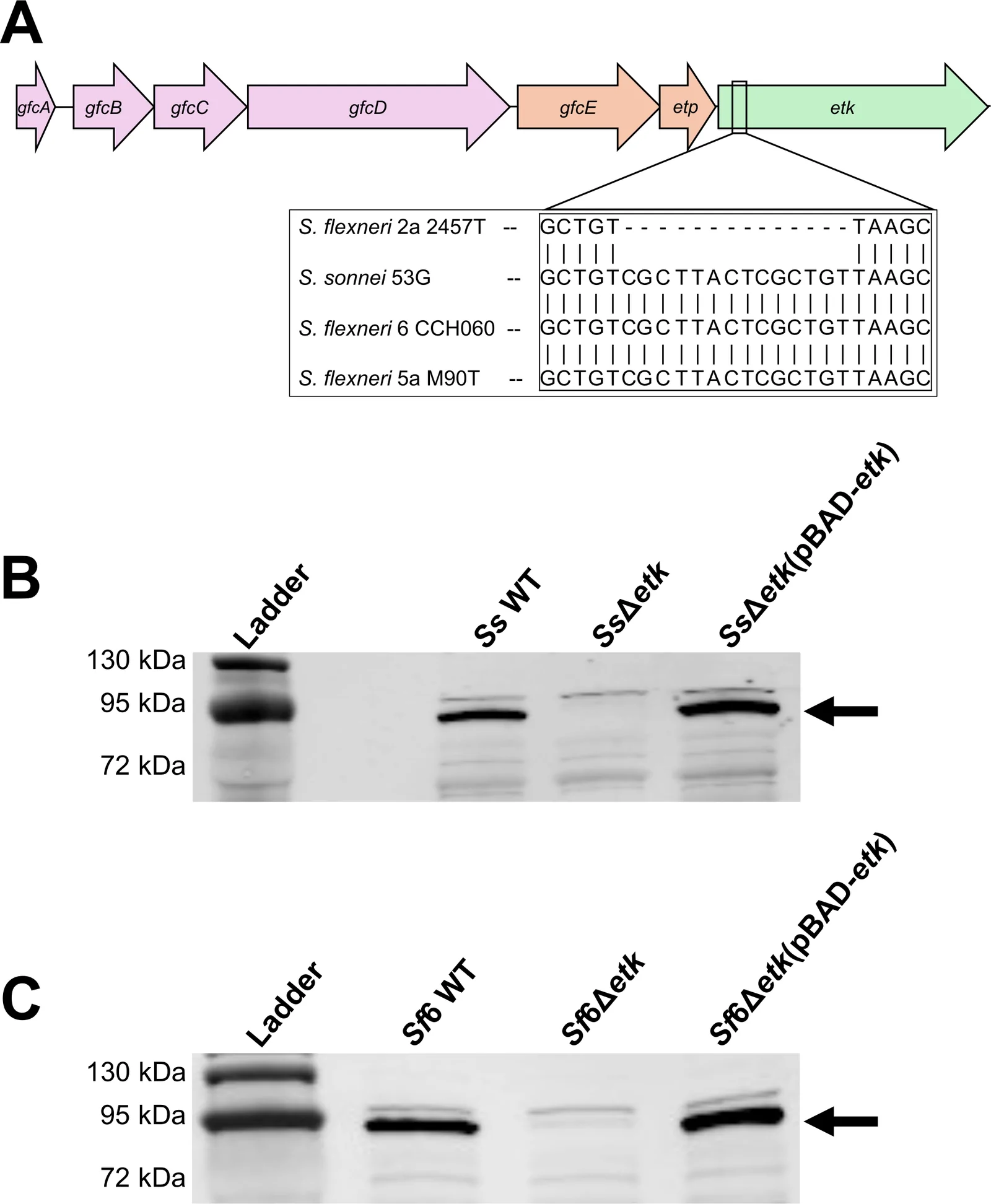
Diagram of the *gfc* operon and Etk expression. Schematic representation of the *gfc* operon necessary for G4C expression as well as the location of the 14-bp deletion reported in *Sf*2a 2457T (**A**). Expression of Etk was determined by Western blot of whole cell lysates of *S. sonnei* strains (**B**) or *Sf*6 strains (**C**) probed with anti-Etk.

Overall, a wide variety of mutations in *etk*, as well as other *gfc* genes, were observed across different *Shigella* species and serotypes. The major difference observed between the *Ss*, *Sf*6, and *Sf*2a *gfc* operons was a mutation in *etk* (Fig. 2A). We generated deletion mutations in *etk* in *Ss* 53G and *Sf*6 CCH060 to assess capsule expression and phenotypic characteristics. A plasmid encoding *etk* under an arabinose-inducible promoter, pBAD-*etk*, was engineered to allow complementation of the *etk* mutant strains. Western blots confirmed that Etk expression was eliminated in the *Ss*∆*etk* and *Sf*6∆*etk* mutants and restored to similar protein levels with the introduction of pBAD-*etk* in both *Ss* (Fig. 2B) and *Sf*6 (Fig. 2C).

### Biochemical confirmation of capsule expression or absence

**Fig 3:**
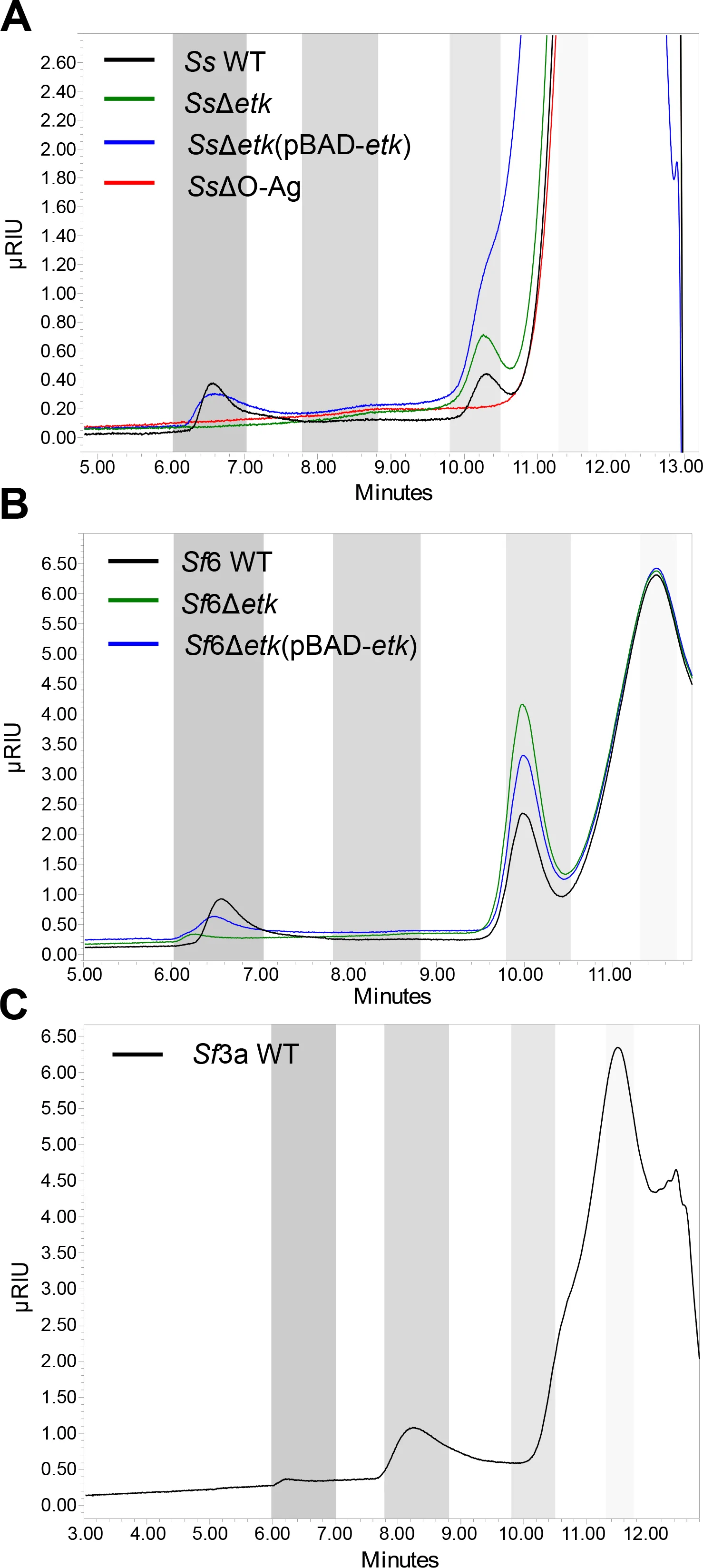
Ultra-performance liquid chromatography–size exclusion chromatography analysis of isolated polysaccharides. Polysaccharides isolated from *Ss* 53G WT, Δ*etk*, ΔO-Ag, and Δ*etk*(pBAD-*etk*) (**A**), *Sf*6 CCH060 WT, Δ*etk*, and Δ*etk*(pBAD-*etk*) (**B**), or WT *Sf*3a J17B (**C**) were separated by UPLC-SEC.

Ultra-high-performance liquid chromatography–size exclusion chromatography (UPLC-SEC) was used to confirm that the presence of an intact *gfc* operon results in capsule expression. Bacterial polysaccharides were purified using phenol extraction followed by mild acid hydrolysis of the lipid A moiety, and the molecular weight distribution was determined using UPLC-SEC. The analysis revealed that *Ss* 53G exopolysaccharide had three peaks representing molecular weight distributions, which include a high-molecular-weight (HMW) peak at ~6.5 min, a medium-molecular-weight (MMW) peak at ~10.5 min, and a low-molecular-weight (LMW) peak at ~11.5 min. The HMW peak was absent from *Ss*∆*etk*, suggesting that this peak is the capsular polysaccharides (Fig. 3A). The *Ss* O-Ag mutant lacked both the HMW- and MMW-sized peaks, suggesting that the MMW peak is LPS O-Ag and that the LMW peak is the core section from LPS. The same trends were observed with *Sf*6 WT and *etk*-mutant derivatives of *Sf*6 CCH060 (Fig. 3B). The HMW peak was restored when *Ss*Δ*etk* (Fig. 3A) and *Sf*6Δ*etk* (Fig. 3B) mutants were complemented with intact *etk* in *trans. Sf*3a J17B, which does not contain an intact *gfc* operon, contained a different O-Ag length composition, as expected, with the peak at ~8.25 min, as well as no HMW polysaccharide peak (Fig. 3C). These results support the consensus that an intact *etk* is required for G4C expression.

### Capsule expression affects colonic epithelial cell invasion levels

**Fig 4:**
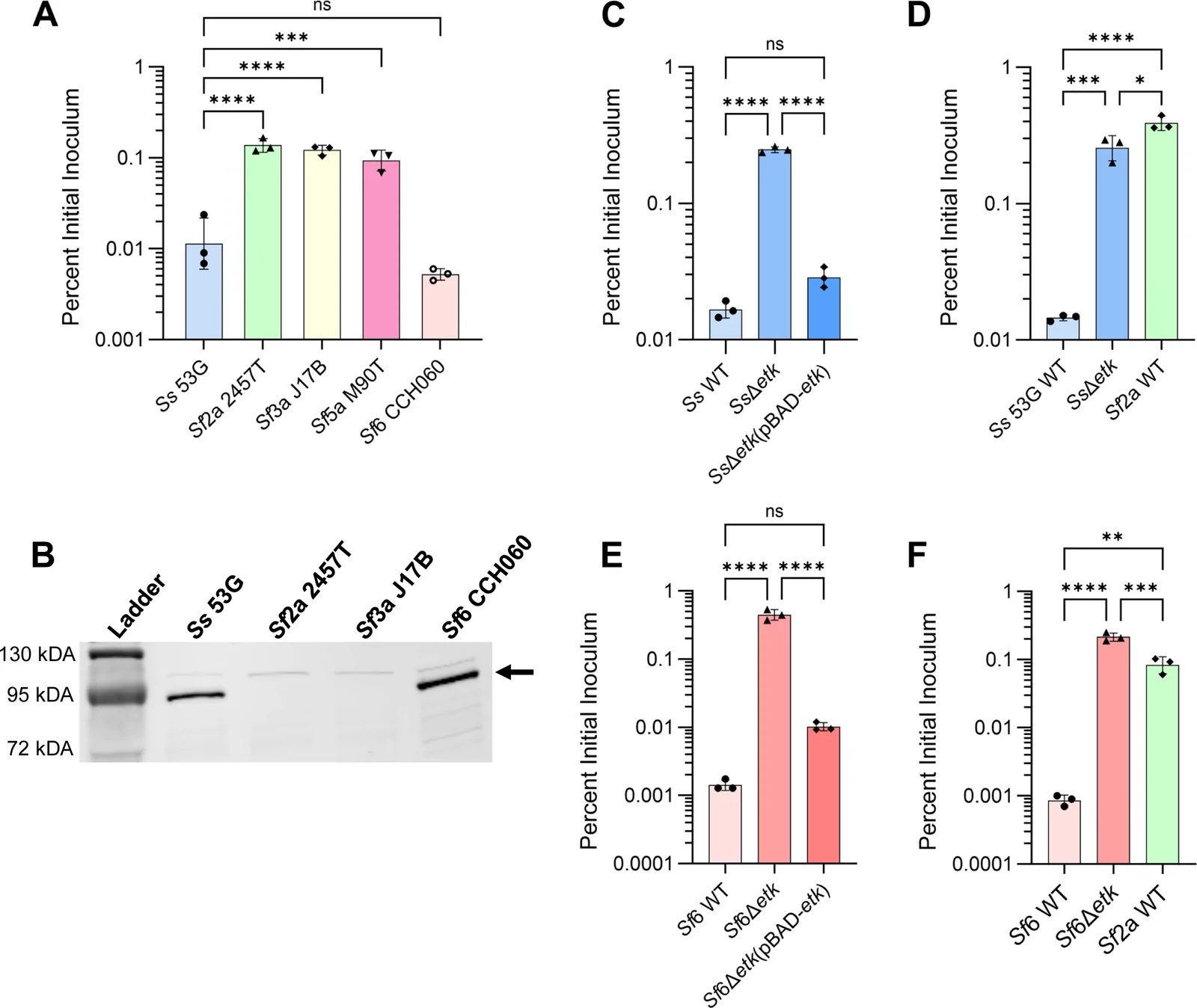
Deletion of *etk* in *Shigella* increases invasion efficiency in HT-29 cells. Human HT-29 cell monolayers were infected with WT *Ss* 53G, *Sf*2a 2457T, *Sf*3a J17B, *Sf*5a M90T, and *Sf*6 CCH060 (**A**), or *Ss* 53G WT, Δ*etk*, Δ*etk*(pBAD-*etk*)*,* or WT *Sf*2a (**C and D**), or *Sf*6 CCH060 WT, Δ*etk*, Δ*etk*(pBAD-*etk*), or WT *Sf*2a (**E and F**) for 90 min, treated with gentamicin for 30 min, washed, and lysed to determine intracellular bacterial counts expressed as percent initial inoculum (CFU recovery). Bacteria were grown either on tryptic soy agar (**A, D, and F**) or in TSB (**C and E**) to mid-log, and *etk* expression was induced in pBAD-*etk* with 0.03% arabinose for 15 min (**E**). Data presented are representative assays from two to three independent experiments. Each dot represents data collected from an individual monolayer. Asterisks indicate statistically significant differences determined using one-way ANOVA with Šídák’s multiple-comparison test. *, *P* < 0.05; **, *P* < 0.01; ***, *P* < 0.001; ****, *P* ≤ 0.0001. Expression of Etk was determined by Western blot of whole cell lysates of WT *Shigella* strains probed with anti-Etk (**B**).

The ability to invade colonic epithelial cells is a hallmark of *Shigella* pathogenesis (48). We have previously demonstrated that *Sf*6 invades epithelial cells significantly less than *Sf*2a and hypothesized that this was due to variation in expression of the type III secretion system (T3SS) effector proteins (47). Using a gentamicin protection assay, we assessed the ability of different *Shigella* strains commonly used in lab settings—*Ss* 53G, *Sf*2a 2457T, *Sf*3a J17B, *Sf*5a M90T, and *Sf*6 CCH060—to invade HT29 cells, a colonic epithelial cell line. *Ss* and *Sf*6 invaded HT29 cells at similar levels to each other yet were significantly lower compared to all the other *Sf* strains tested (Fig. 4A). Lack of Etk protein production, assessed by Western blots, correlated with higher invasion levels (Fig. 4B). We hypothesized that the observed lower invasion levels of *Ss* and *Sf*6 compared to other *Sf* strains were due to capsule expression.

Deletion of *etk* in *Ss* 53G (Fig. 4C) and *Sf*6 CCH060 (Fig. 4E) resulted in a significant increase in HT29 cell invasion, comparable to invasion levels of *Sf*2a 2457T (Fig. 4D and F). Complementation of *Ss*Δ*etk* and *Sf*6Δ*etk* with intact *etk* restored invasion rates to their respective parental WT reduced levels.

**Fig 5:**
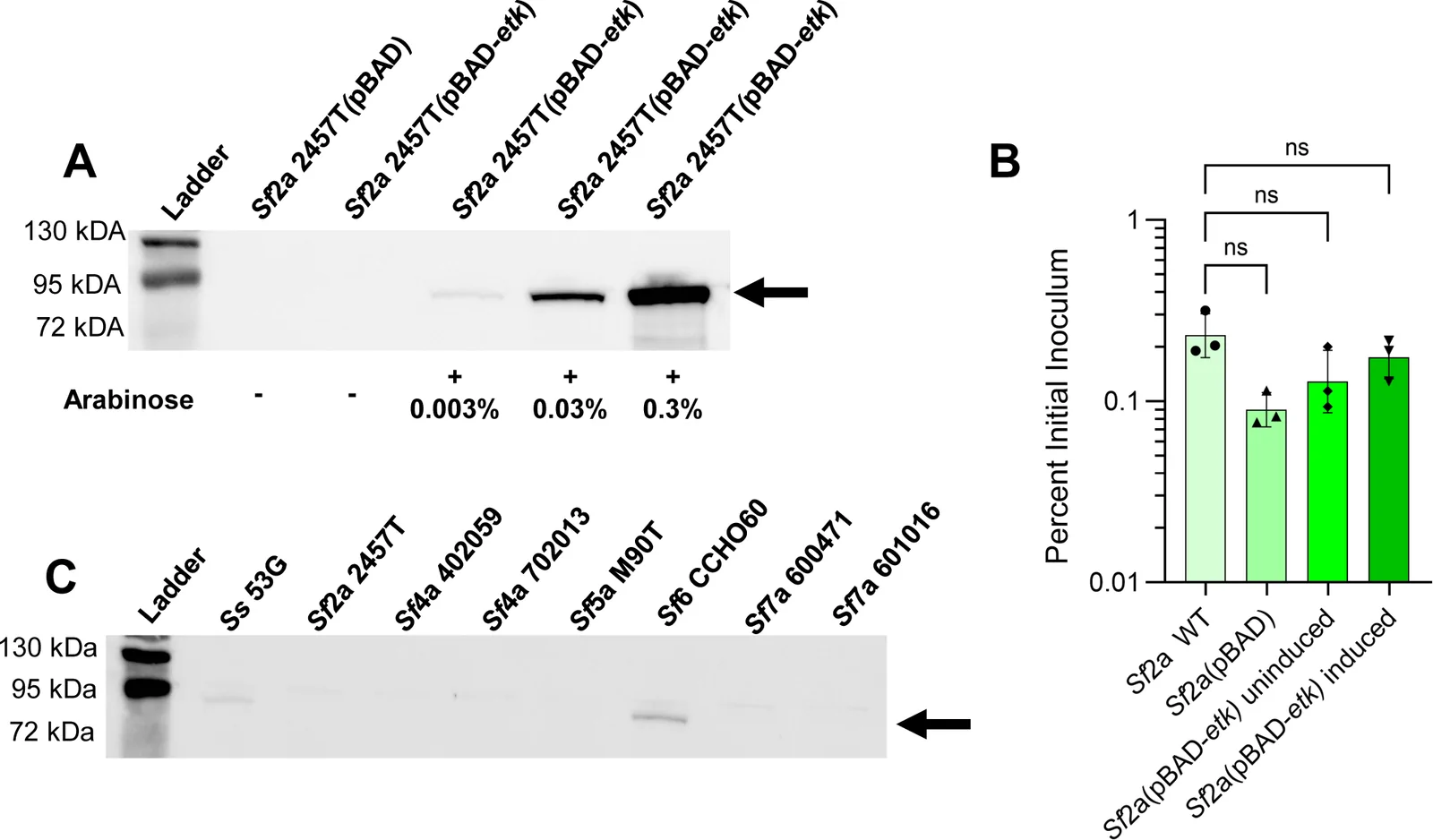
*Sf*2a, *Sf*4a, and *Sf*5a do not express the *gfc* operon. Expression of Etk was determined by Western blot of whole cell lysates of WT *Sf*2a 2457T, or *Sf*2a 2457T(pBAD), or *Sf*2a(pBAD-*etk*) grown in TSB to mid-log phase with *etk* induction induced with 0.003%–0.3% arabinose for 15 min, probed with anti-Etk (**A**). Human HT-29 cell monolayers were infected with WT *Sf*2a 2457T, WT *Sf*2a 2457T, or *Sf*2a 2457T(pBAD), or *Sf*2a(pBAD-*etk*), both uninduced and induced for 90 min, treated with gentamicin for 30 min, washed, and lysed to determine intracellular bacterial counts expressed as percent initial inoculum (CFU recovery) (**B**). Bacteria were grown in TSB to mid-log, and *etk* expression was induced in pBAD-*etk* with 0.03% arabinose for 15 min. Data presented are representative assays from two independent experiments. Each dot represents data collected from an individual monolayer. Statistically significant differences were determined using one-way ANOVA with Šídák’s multiple-comparison test. Expression of Etk was determined by Western blot of whole cell lysates of *Ss*, and Sf2a, 4a, 5a, 6, and 7a strains probed with anti-Etk (**C**).

The intact *etk* gene was introduced into *Sf*2a 2457T by complementation using plasmid pBAD-*etk*, and Etk expression was confirmed by Western blot (Fig. 5A). Surprisingly, the invasion phenotype of *Sf*2a 2457T (pBAD-etk) remained at the high level exhibited by the WT strain (Fig. 5B). To determine if this phenotype was strain dependent, invasion levels were compared between the commonly used lab strain *Sf*2a 2457T and two *Sf*2a clinical isolates from geographically diverse regions (Fig. S1A). The clinical isolates had similar invasion levels to *Sf*2a 2457T, and, as with 2457T, introduction of intact *etk* did not alter the invasion efficiency. The presence of intact Etk alone was not sufficient to reduce the invasion phenotype in *Sf*2a, suggesting that G4C was not expressed.

### Additional regulatory elements affect G4C expression in *S. flexneri*

*Sf*2a contains fully intact versions of the first six genes in the *gfc* operon. The lack of a decreased epithelial cell invasion phenotype following the introduction of an intact *etk* gene, the seventh gene in the *gfc* operon, suggested that additional regulatory factors affected G4C expression. We examined additional *Sf* serotypes and strains that we identified as containing intact *gfc* operons. *Sf*5a strain M90T, which has a high invasion efficiency yet contains an intact *gfc* operon, and representative isolates of *Sf*4a and *Sf*7a, which all contain intact *gfc* operons, were tested for Etk expression by Western blot. All of these strains lacked Etk protein expression (Fig. 5C). This indicates that there are additional factors required for G4C expression.

**Fig 6:**
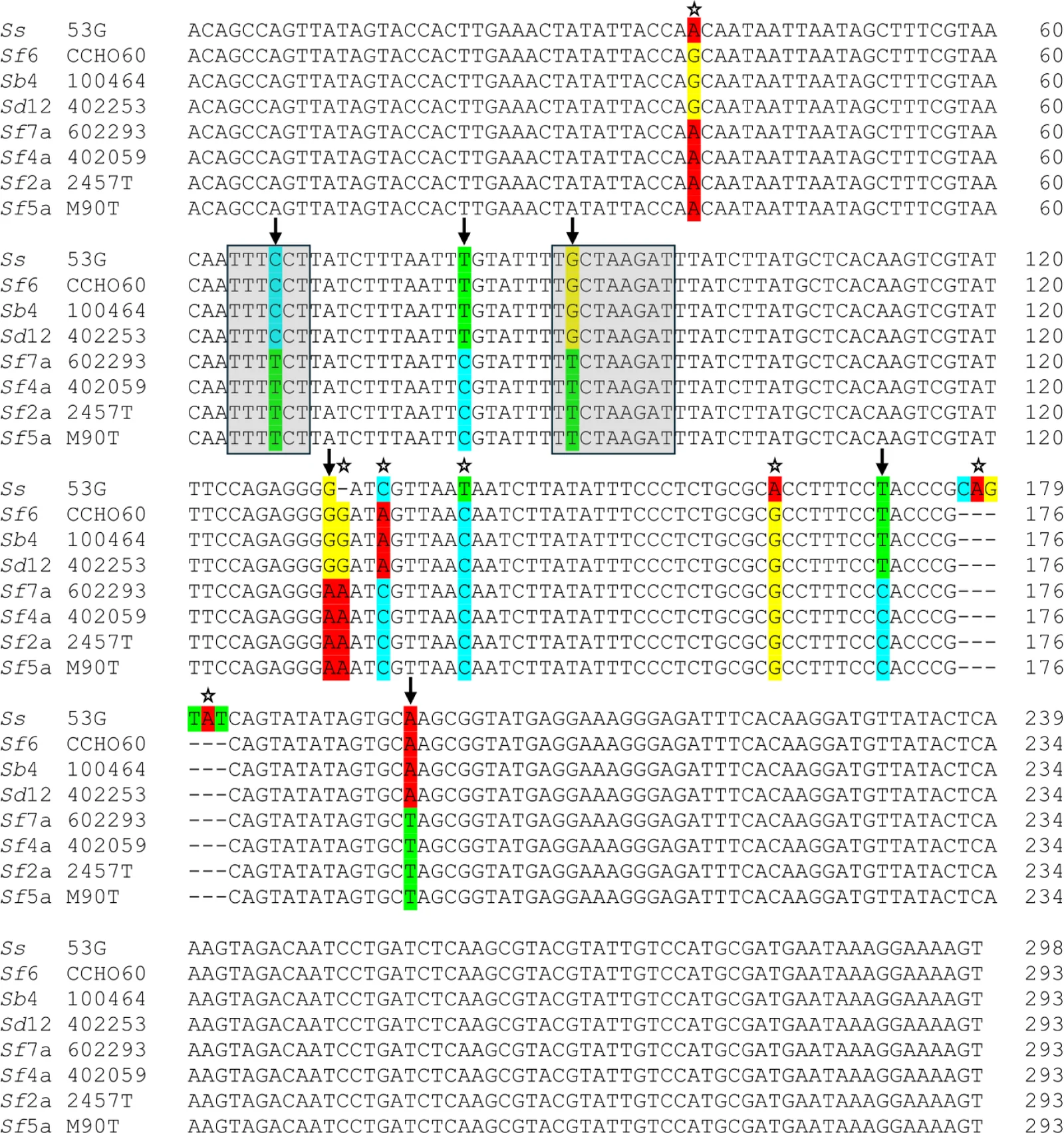
Specific sequence differences are observed between G4C-expressing and non-expressing strains. The *gfc* upstream regions, including 298 bp in *Ss* 53G and 293 bp in other strains, were aligned using ClustalOmega. Differences in nucleotides are highlighted. Regions with differences between expressing and non-expressing strains are marked with a vertical arrow. Regions with differences between *Ss* 53G and other expressing strains are marked with a star. The −35/−10 promoter regions were predicted using the SoftBerry prediction tool and are marked with a gray box.

In order to determine if there were sequences upstream of the *gfc* operon that affect expression, an alignment of the entire 293–298 bp upstream regions of strains that produce Etk protein (*Ss* 53G, *Sf*6 CCH060, *Sb*4 100464, and *Sd*12 402253) and lack Etk production (*Sf*2a 2457T, *Sf*4a 402059, *Sf*5a M90T, and *Sf*7a 602293) was performed. Six nucleotides were identified that differ between all Etk-producing strains and all Etk-non-producing strains (Fig. 6). Additionally, the *Ss* upstream region had 11 additional nucleotides across six different regions, including 1 deletion, 4 substitutions, and 6 insertions, that differed from all the other Etk-producing strains.

**Fig 7:**
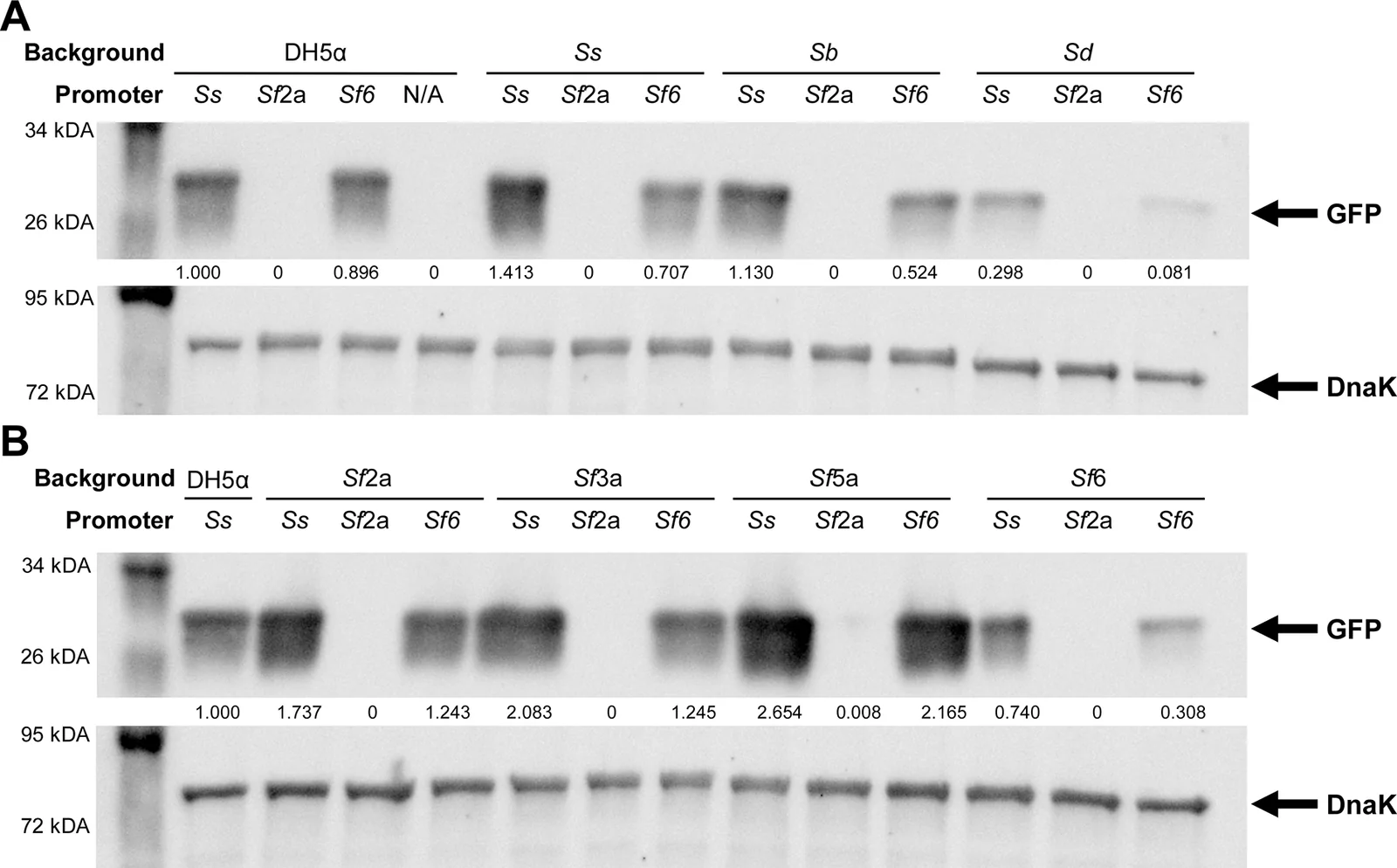
The *gfc* native promoters for Ss53G and Sf6CCHO60 both drive expression of GFP, while the promoter for Sf2a does not. A promoterless *gfp* gene was cloned downstream of the promoter and 5′ UTR of *Ss* 53G, *Sf*2a 2457T, and *Sf*6 CCH060. These constructs were electroporated into different *Shigella* background strains: DH5α, *Ss*, *Sb*, and *Sd* (**A**) and *Sf*2a, *Sf3a*, *Sf5a*, and Sf6 (**B**). Expression of GFP and DnaK, as a control, in whole cell lysates of different background strains containing the three plasmid constructs or no plasmid (N/A) was probed with anti-GFP. Densitometry (ImageJ) of GFP was performed and was expressed relative to GFP from DH5α(pZA-P_SS_:*gfp*) to normalize.

The upstream regions from *Ss* 53G, *Sf*6 CCH060, and *Sf*2a 2457T were cloned individually into a promoterless plasmid with green fluorescent protein (GFP) to determine native *gfc* promoter functionality. The resulting plasmids—pZA-P_SS_:*gfp*, pZA-P_SF6_:*gfp*, and pZA-P_SF2a_:*gfp*—were transformed into *Ss*, *Sf*2a, *Sf*3a, *Sf*5a, *Sf*6, *Sb*, and *Sd* background strains and assessed for GFP activity. In all background strains, the *Ss* and *Sf*6 *gfc* promoters drove GFP expression. In contrast, the *Sf*2a promoter fusion resulted in no detection of GFP in any background strain (Table 3) (Fig. S2). GFP production, or lack thereof, was confirmed by Western blot in all strains (Fig. 7A and B). In all background strains, GFP production was detected from the *Ss* and *Sf*6 *gfc* upstream regions, and no production was observed from the *Sf*2a upstream region, suggesting that one or more of the six nucleotides are necessary for expression of the *gfc* native promoter. Additionally, while GFP was not detected visually in *Sb* and *Sd*, GFP protein was observed in both strains containing the plasmids with *Ss* or *Sf*6 promoter-driven GFP when examined by Western blot. It was further noted that the *Ss* upstream promoter sequences drove a higher level of GFP compared to the *Sf*6 region in all strains.

**TABLE 3:** GFP production from different *gfc* promoters^*a*^

Background strain	pZA-P_SS_:*gfp*	pZA-P_SF2a_:*gfp*	pZA-P_SF6_:*gfp*
DH5α	++	−	++
*Sf*2a	+++	−	+++
*Sf*3a	+++	−	++
*Sf*5a	++++	−	++
*Sf*6	+	−	+
*Ss*	+++	−	+++

^*a*^ Fluorescence was graded visually on a scale from −, +, ++, +++, and ++++, where the peak fluorescence was given a ++++ rating.

### Recent *S. sonnei* clinical isolates express Etk

**Fig 8:**
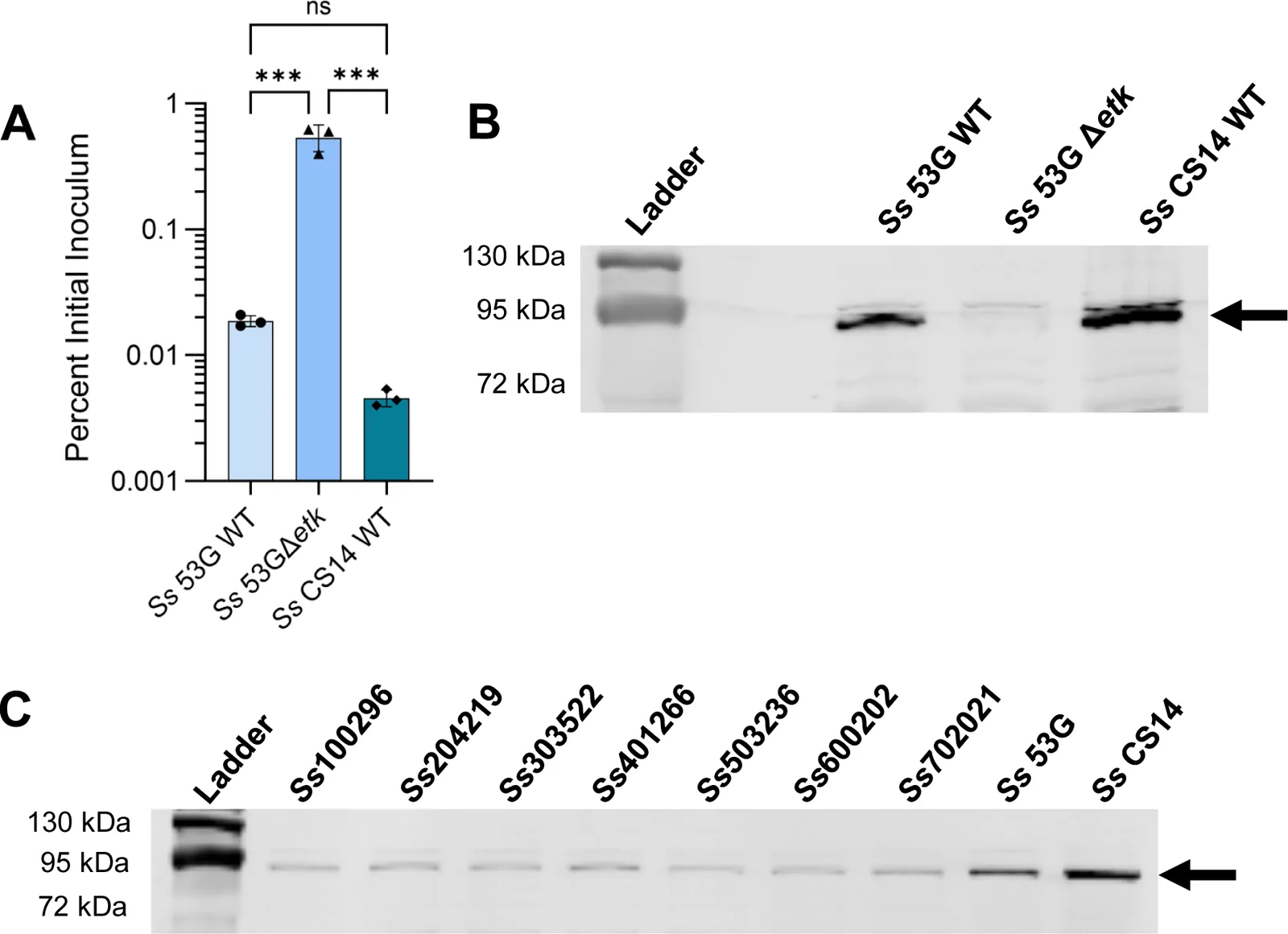
*Ss* clinical isolate express a G4C. Human HT-29 cell monolayers were infected with WT *Ss* 53G, Ss 53GΔ*etk*, and clinical isolate *Ss* CS14 for 90 min, treated with gentamicin for 30 min, washed, and lysed to determine intracellular bacterial counts expressed as percent initial inoculum (CFU recovery) (**A**). Each dot represents data collected from an individual monolayer. Asterisks indicate statistically significant differences determined using one-way ANOVA with Šídák’s multiple-comparison test. ***, *P* < 0.001. Expression of Etk was determined by Western blot of whole cell lysates of WT *Ss* 53G, Δ*etk*, and clinical isolate *Ss* CS14 (**B**) and in WT *Ss* clinical isolates (**C**) probed with anti-Etk.

It has been previously reported that different lineages of *Ss*, and specifically recent clinical isolates, vary in their *gfc* operon transcript expression levels (49). *Ss* has a naturally unstable pINV virulence plasmid, which is lost in lab settings at a high rate (50, 51). This results in difficulty in accurately measuring the invasion levels of clinical isolates of *Ss*. We have previously engineered a stabilized pINV version of *Ss* 53G, named 53G-SP, which has been used throughout these studies (50). In order to determine if the low invasion level of *Ss* 53G-SP was observed in other strains, we utilized the clinical isolate CS14, which contains a naturally stabilized pINV (51). We observed that both *Ss* 53G and *Ss* CS14 invaded HT-29 cells at a significantly lower level compared to *Sf*2a (Fig. 8A). *Ss* CS14 and *Ss* 53G both expressed the Etk protein (Fig. 8B). We also confirmed production of Etk for seven geographically diverse isolates of *Ss* (Fig. 8C).

### *S. dysenteriae* and *S. boydii* express a G4C

**Fig 9:**
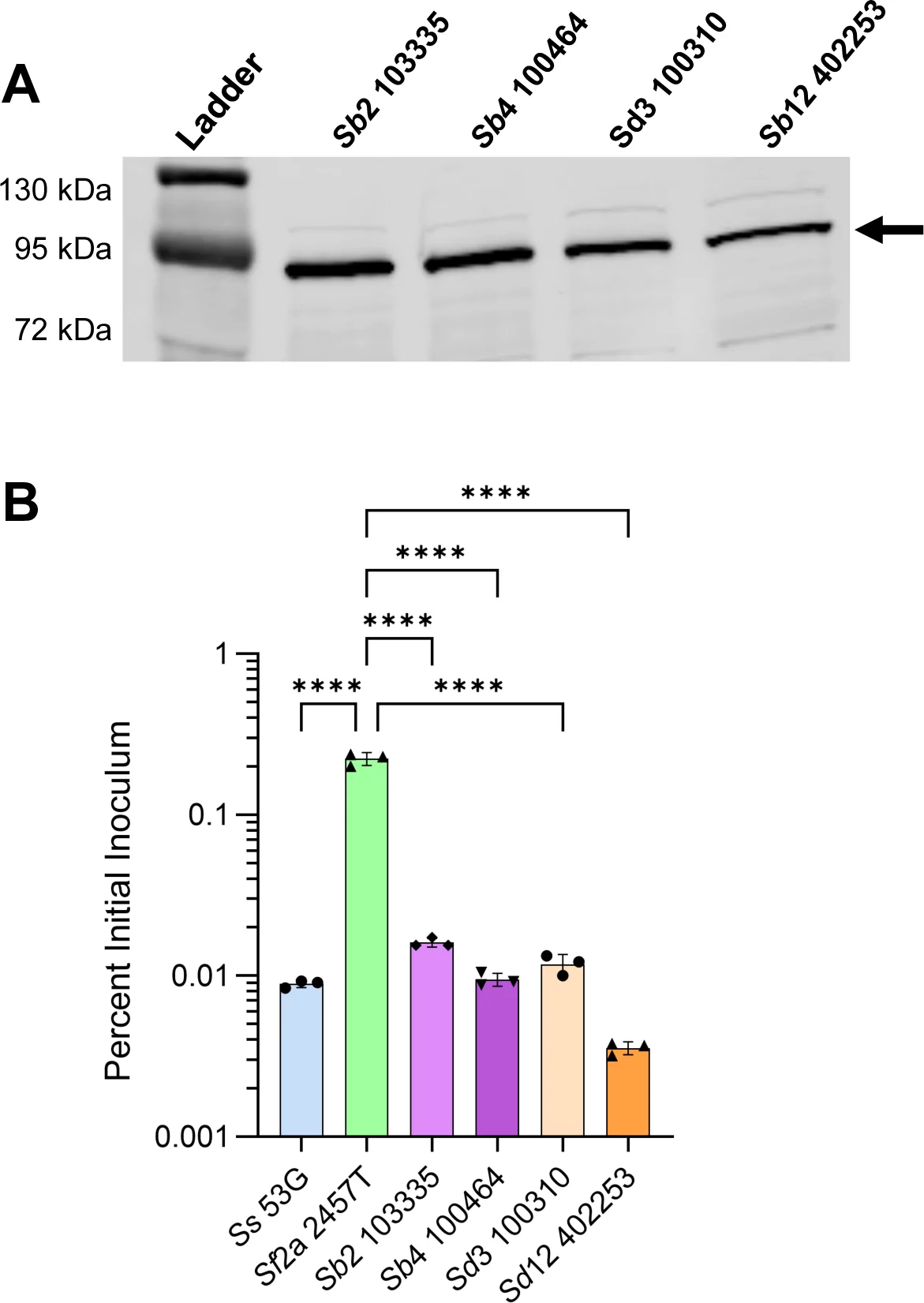
*S. boydii* and *S. dysenteriae* express a G4C. Etk expression in whole cell lysates of WT *Sb* and *Sd* clinical isolates, prepared from inocula used for invasion assays in panel B, was determined by Western blot probed with anti-Etk (**A**). Human HT-29 cell monolayers were infected with WT *Ss* 53G, *Sf*2a 2457T, WT *Sb,* and Sd clinical isolates (**B**) grown on tryptic soy agar for 90 min, treated with gentamicin for 30 min, washed, and lysed to determine intracellular bacterial counts expressed as percent initial inoculum (CFU recovery). Each dot represents data collected from an individual monolayer. Asterisks indicate statistically significant differences determined using one-way ANOVA with Šídák’s multiple-comparison test. ****, *P* ≤ 0.0001.

*Sb* and *Sd* are reported at lower numbers than *Ss* and *Sf* in epidemiological studies but remain clinically relevant (1, 3, 9). We confirmed Etk expression in two clinical isolates, each of *Sb* and *Sd* (Fig. 9A). The invasion levels of two clinical isolates of *Sb* and *Sd* (Fig. 9B) were significantly less than *Sf*2a and were at similar levels to *Ss*. These data support the presence of a G4C in both *Sb* and *Sd*.

## DISCUSSION

Capsule expression by bacteria has been shown to have profound effects on virulence, including host interactions, antimicrobial resistance, and immune evasion (52–55). The identification of a G4C on *Ss* was previously shown to affect important pathogenic processes, including host cell invasion, the hallmark of *Shigella* pathogenesis (30, 35). The expression of G4C in *Ss* reduced invasion efficiency compared to *Sf*2a, which contains a 14-bp deletion in *etk*, the terminal gene in the seven-gene operon required for G4C expression. Our recent finding of reduced invasion by *Sf*6 strains (47) prompted us to consider the role of G4C in this serotype, as well as to search for G4C expression in other *Shigella* species and serotypes. Genomic analysis of 1,219 *Shigella* clinical isolates from the GEMS study revealed that the presence of an intact *gfc* operon was highly conserved within *Ss*, *Sf*6, *Sd*, and *Sb*. This is the first large-scale analysis of *Shigella* clinical isolates assessing the presence and integrity of the *gfc* operon and shows that current circulating strains from these species and serotypes are likely to express a capsule.

We report that all *Sf*2a isolates (*n* = 228), the most prevalent *Sf* serotype (3, 9, 56, 57), had genetically intact and highly identical (99.98% identity) versions of the first six genes in the *gfc* operon and harbored the same 14-bp deletion in *etk*. There was a variety of genetic mutations identified across different *Sf* serotypes, as well as within the same serotype. Like *Sf*2a, 100% of *Sf*2b isolates contained the 14-bp *etk* deletion, whereas other *Sf* serotypes did not. Additionally, there were strains both with and without the 14-bp deletion within the same serotype, such as *Sf*4a. Overall, there was a variety of different mutations across the *gfc* operon that resulted in an expected loss of function.

Interestingly, a genetically intact *gfc* operon was observed in a high percentage of *Sf*4a (54%), as well as *Sf*7a (39%) isolates. The presence of an intact *gfc* operon in *Sf*, other than *Sf*6, has not been previously recognized. However, these strains contained SNPs upstream of the *gfc* coding region that resulted in no *gfc* expression. This demonstrates that the presence of intact *gfc* coding regions is not sufficient to predict G4C expression, emphasizing the importance of pairing genomic analysis alongside mechanistic analysis.

**Fig 10:**
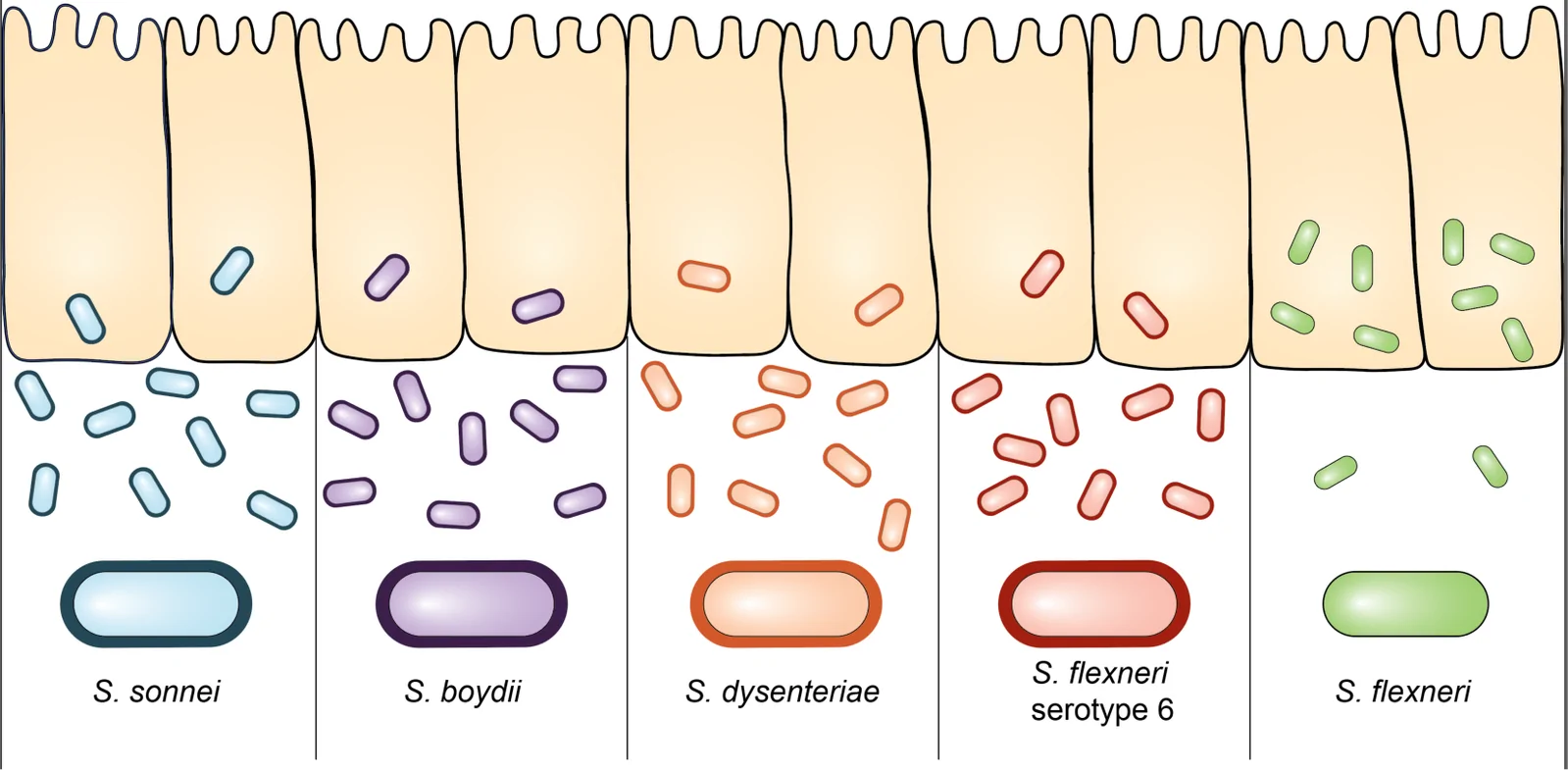
Diagram of different *Shigella* species for capsule presence and invasion phenotype. *Ss*, *Sb*, *Sd*, and *Sf*6 express a capsule, whereas other *Sf* serotypes do not. All strains that express a capsule invade at a lower efficiency compared to strains that lack a capsule.

We previously reported the genomic divergence of *Sf*6 from other *Sf* (46), as well as its proximity to *Sb* strains, which we now report also have a conserved intact *gfc* operon. We also demonstrate that *Sb* isolates (71/71), as well as *Sd* (58/60), express Etk and invade epithelial cells at a low, capsule-positive, level, lending supporting evidence for the expression of a G4C on the surface. Overall, this expands upon the knowledge regarding the presence of capsule and resulting phenotype across a wider range of strains than previously recognized (Fig. 10). It should be noted that our analysis does not include any *Sd* serotype 1 strain; further examination of the capsule previously reported in *Sd*1 (42, 43) and its presence in *Sd*1 isolates is needed.

All strains that expressed a G4C, including *Ss*, *Sf*6, *Sb*, and *Sd*, exhibited lower epithelial cell invasion levels compared to the *Sf* strains that lacked a G4C and the *etk* mutant derivatives of *Ss* and *Sf6*. Restoration of WT invasion levels to mutants by complementation, along with supporting UPLC-SEC results, confirmed the role of the capsule in invasion efficiency. It has been hypothesized that capsule expression directly impacts epithelial cell invasion by steric hindrance of the T3SS injectosome, which is necessary to induce invasion (30). Our data expand upon this observation and suggest that all *Shigella* strains that express a capsule invade epithelial cells with lower efficiency than strains that lack a capsule. Combined with genomic data, these findings contribute to a greater understanding of key differences between *Shigella* species, serotypes, and strains.

Genomic analysis underscored the role of *etk* as a major target of mutations in various *Shigella* strains. Biochemical analysis using UPLC-SEC confirmed that deletion in *etk* resulted in loss of capsule expression in *Ss* and *Sf*6. While complementation with intact *etk* in *Ss* and *Sf*6 mutants restored capsule expression and the reduced invasion phenotype, this plasmid did not confer the expected decreased invasion level when introduced into WT *Sf*2a strain 2457T or *Sf*2a clinical isolates.

Comparison of *gfc* upstream regions from Etk expression strains to Etk non-expressing strains showed conserved regions that differed between the two groups. Six conserved nucleotides were identified in all G4C-expressing strains that were different in non-expressing strains. Additionally, cloning the upstream promoter regions behind *gfp* showed that these differences were sufficient to abolish GFP expression in the *Sf*2a promoter, regardless of the background strain it was transformed into. Interestingly, in all background strains, the *Ss* 53G promoter and 5′ UTR region resulted in more GFP production compared to the *Sf*6 CCH060 promoter and 5′ UTR region, even in *Sf*6 CCH060 itself. These results indicate that specific nucleotide differences in this region can alter expression of the *gfc* operon. Additionally, the differences observed between background strains using the same reporter plasmid indicate that there may be differential expression of regulatory components. The regulation of the G4C is known to include the Ler transcription factor in EHEC. However, this transcription factor is not present in *Shigella*, and there are no published studies on the regulation of G4C in *Shigella* (54). Future research into the conditions that enable capsule expression in *Shigella*, as well as differences observed between strains, will elucidate key steps during pathogenesis that could be impacted by capsule expression during human infection.

While *Sf*2a 2457T encodes a truncated, non-functional Etk and therefore no capsule, it expresses a bimodal chain length LPS O-Ag (58–60). *Sf*2a 2457T contains the gene *wzz*_SF_, also referred to as *wzzB*, located on the chromosome that results in a short O-Ag chain (11–17 O-Ag repeat units), as well as a *wzz*_pHS2_ located on the pHS-2 plasmid that results in a longer O-Ag chain (>90 O-Ag repeat units) (58). However, unlike the G4C in *S. sonnei*, the presence of this very long O-Ag chain length does not impact HeLa cell invasion (58). Additionally, *Sf*5a also expresses a bimodal LPS O-Ag length dependent on the glucosylation state of the O-Ag (61). The fact that these strains contain the genes necessary for a capsule but lack their expression suggests that at one point they too may have expressed a capsule. It is possible that the capsule was replaced with the bimodal O-Ag currently utilized that allows all lengths of O-Ag to be expressed exclusively on lipid A core. We hypothesize that these serotypes and strains evolved away from capsule expression toward the use of a single export system for all lengths of O-Ag in order to increase metabolic efficiency.

Interestingly, analysis of extracted polysaccharides from *Sf*6Δ*etk* demonstrated a small HMW polysaccharide peak, but to a lesser degree than in the WT strain. This supports the hypothesis that Etk is involved in the export of the HMW O-Ag but not the polymerization of HMW O-Ag. We hypothesize this population of HMW O-Ag is likely trapped in the periplasm and released during cell lysis in sample preparation. We show for the first time that *Sf*3a J17B, which does not contain an intact *gfc* operon, has a similar small HMW peak compared to that seen in *Sf*6Δ*etk*. We hypothesize that just as capsule O-Ag (HMW) polysaccharides utilize a dedicated *gf*c-encoded export system compared to LPS O-Ag (MMW), the two different groups of O-Ag also require dedicated WzzB proteins, which is the chain length determinant. Additionally, *Sf*3a J17B expresses a WzzB homolog necessary to polymerize O-Ag to the capsule length. Why *Sf*3a J17B synthesizes this form of O-Ag when it cannot be exported to the surface is unknown, and is the subject of further research.

In addition to its role in pathogenesis, the expression of G4C could impact the performance of live attenuated vaccines. It has been demonstrated that different O-Ag lengths on a membrane-vesicle vaccine (generalized modules for membrane antigens) did not alter anti-LPS titers for both *Ss* and *Sf*6 (44, 45). Alternatively, capsule expression could hinder immune responses to other surface antigens that may be important for inducing an optimal protective response. Furthermore, the fact that capsule expression affects *Shigella*’s interaction with epithelial cells suggests that other aspects of host interaction could be impacted by capsule expression as well, such as cell-to-cell spread or interactions with antigen-presenting cells. Overall, the impact of G4C expression on vaccine strains warrants further investigation.

## MATERIALS AND METHODS

### Bacterial strains and growth conditions

Strains (Table S1) were grown in tryptic soy broth (TSB) or tryptic soy agar (TSA) (BD Difco). Congo red (Sigma-Aldrich, St. Louis, MO) was added at a final concentration of 0.01% (wt/vol) and supplemented with 0.005% guanine to make CR-TSA plates. When indicated, strains were grown on M9 salt minimal media (62). Antibiotics were added at the following concentrations: carbenicillin, 100 μg/mL;, kanamycin, 50 μg/mL; and chloramphenicol, 12.5 μg/mL.

### Construction of mutants

Mutations in *etk* were engineered using the Lambda-Red system as described (63). Primers 1F and 1R (for *etk* in 53G) or 2F and 2R (for *etk* in CCH060) were used to amplify the kanamycin resistance gene in pKD4 (63). Mutagenic PCR products were electroporated into the corresponding *Ss* or *Sf*6 strain, and resultant antibiotic-resistant colonies were identified. The kanamycin resistance gene was then eliminated using a helper plasmid pCP20 (63). Unmarked gene deletions in the resultant strains *Ss*Δ*etk* and *Sf*6Δ*etk* were confirmed by PCR and sequencing.

### Construction of plasmids

Complementation plasmid pBAD-*etk* was created by amplifying the *etk* gene from *Ss*53G with primers 3F and 3R and ligating into pBAD; all primers and plasmids are listed in Table S2. Plasmid pZA112-trc (64) was digested with BamHI and EcoRI, treated with mung bean nuclease, and self-ligated to create promoterless pZA(−)*trc. Shigella gfc* native promoters were amplified from designated strains using primers *ymcD*_F_NheI and G4C_GFP_R. GFP was amplified from pGEN206-GFP (65, 66) using primers G4C_GFP_F and GFP_R_AvrII. Both PCR products were purified and mixed equally in overlapping PCR reactions to create promoter-GFP fusions. The GFP fusion PCR products were digested with NheI and AvrII and ligated into pZA(-trc). Primers pZA_001 and pZA_002 were used to screen for positive clones and confirmed via nanopore sequencing (Quintara Biosciences). All enzymes and DH5α-competent cells were purchased from New England Biolabs (Beverly, MA).

### Genomic analysis of *gfc* operon

The genomic content of all *Shigella* species isolates from Bengtsson et al. (26) and of the identified *Sf* serotypes was analyzed. Using BLAST (67, 68), we compared the *etk* gene or the *gfc* gene cluster with >1200 genomes from the GEMS (2) to identify the prevalence of the genes and associated mutations. The genetic regions were compared using MUSCLE (69), and the mutations were cataloged between different *Shigella* species and serotypes.

Genetic variation in *S. flexneri* was examined using a customized genomic analysis pipeline. Briefly, contigs from individual samples (FASTA format) were aligned to the *S. sonnei Ss*046 reference *gfc* operon (GenBank CP000039.1) using minimap2 (2.24-r1122) (70) with the option “-ax asm10 -eqx.” The resulting alignments (BAM format) were filtered to retain reads overlapping the *gfc* operon. These aligned reads were parsed using custom Python scripts leveraging the pysam (v0.23.1) (71) library to identify genetic variants, including SNPs, short and long insertions/deletions, and gaps (regions not covered by any contigs). Variants were exported in variant calling format using a customized Python script and annotated using SnpEff (v5.2f) (72). A custom SnpEff database was built for the *gfc* operon region in the *Ss*046 genome.

The promoter regions were aligned using ClustalOmega 1.2.2 (73). Predicted promoter sequences were determined using the SoftBerry BPROM prediction tool (74).

### Polysaccharide sample preparation

Polysaccharide samples were prepared with the phenol-water method as described (75). Strains were subcultured in 50 mL TSB to an OD_600_ of 0.8–1.0. Arabinose (0.03%) was added for the final ~20 min for strains containing pBAD-etk. Samples were pelleted, resuspended in 500 µL phosphate-buffered saline (PBS), boiled for 5 min, treated with 0.5 µg/µL Proteinase K, and incubated at 60°C overnight. Five hundred microliters of saturated phenol was added and incubated for 1 h at 70°C. Samples were centrifuged for 30 min at 10,000 × *g*, the upper phase collected, mixed with two volumes of absolute ethanol, incubated for 1 h at −70°C, centrifuged at 12,000 × *g* for 30 min at RT, and lyophilized. Pellets were resuspended in 1% acetic acid and boiled for 1.5 h, and the pH was adjusted to ~6.5 to 7.5 with 1 M ammonium hydroxide. Samples were centrifuged at 4°C for 35 min, and the supernatant dialyzed against water overnight at 4°C using a 3.5K MWCO cassette (Thermo Scientific) with three water changes.

### Biochemical determination of capsule by UPLC-SEC

Polysaccharide dialysates were analyzed by UPLC-SEC using a BioZen 1.8 µm SEC-3 column (Phenomenex, California) run at 0.3 mL/min on an Acquity UPLC (Waters, Massachusetts) in 1× PBS/0.02% sodium azide, pH 7.4, with detection by refractive index (RI) on a 2414 RI detector (Waters).

### Cell culture and gentamicin protection assay

Human HT-29 cells were cultured and used for gentamicin protection assays as described (47), using gentamicin at 75 µg/mL.

### GFP fluorescent imaging

Strains were grown on M9 salt minimal media, and images were taken on a Spectroline Ultraviolet Transilluminator. Four blinded researchers rated the degree of fluorescence on a 1–4 scale, and results were averaged.

### Etk-specific antibodies

Etk-specific polyclonal antibody was produced in rabbits as a contract (GenScript) using the 281-amino acid cytoplasmic domain of Etk from *S. sonnei* 53G (WP_000208650) expressed as a recombinant protein.

### Protein expression

Whole cell bacterial lysates were prepared for Western blot analysis as follows. Innocula from the correlating gentamicin protection assay or overnight bacteria grown on TSA + CR were collected in PBS and normalized for bacterial numbers by OD_600_. Suspensions were mixed 1:1 with 2× Laemmli sample buffer with 5% β-mercaptoethanol (Bio-Rad). Proteins were separated on a 12% Mini-Protean TGX Precast Gel (Bio-Rad) and transferred to a PVDF membrane. The membrane was blocked in 10% (wt/vol) non-fat milk in 1× Dulbecco’s PBS and incubated with α-Etk, monoclonal rabbit α-DnaK (Invitrogen), or monoclonal mouse α-GFP (Santa Cruz Biotechnology). Following washing, the membranes were incubated with secondary antibody goat α-rabbit 680 nm (Invitrogen) or goat α-mouse 700 nm (Invitrogen), and proteins were visualized using the LI-COR Odyssey Laser Scanner or BioRad ChemiDoc MP imaging system.

## Data Availability

All clinical isolates have short-read sequences from reference 26, as indicated in Table S1, and have previously been deposited in the European Nucleotide Archive (https://www.ebi.ac.uk/ena/) under project accession number PRJEB45383. Publicly available sequences were downloaded from GenBank (https://www.ncbi.nlm.nih.gov/genbank/).

## SUPPLEMENTAL MATERIAL

Fig. S1 msphere.00354-26-s0001.tif Sf2a HT29 invasion assay.

Fig. S2 msphere.00354-26-s0002.tiff GFP agar plates.

Supplemental material msphere.00354-26-s0003.docx Tables S1 and S2; legends for Fig. S1 and S2.

ASM does not own the copyrights to Supplemental Material that may be linked to, or accessed through, an article. The authors have granted ASM a non-exclusive, world-wide license to publish the Supplemental Material files. Please contact the corresponding author directly for reuse.
