# Supplementary material for: Expanded prevalence of group 4 capsule expression and its genetic requirements in a broad array of Shigella strains
Source: mSphere. 2026 Aug 12;11(9):e00354-26. doi: 10.1128/msphere.00354-26 (PMC13621845; doi:10.1128/msphere.00354-26)
Supplement: Supplementary file 1 [file msphere.00354-26-s0003.docx]

**Supplemental Materials**

Table S1. Strains used in this study

Table S2. Primers and plasmids used in this study

Figure S1. Expression of Etk in clinical isolates of *Sf*2a strains does not reduce invasion efficiency in HT-29 cells

Figure S2. The *gfc* native promoter for *Ss* 53G and *Sf*6 CCHO60 both express GFP while the promoter for *Sf*2a does not

| **Supplemental Table 1: Strains used in this study** | | | | |
| --- | --- | --- | --- | --- |
| **Species** | **Serotype** | **Strain name** | **Country of origin** | **Reference** |
| *Shigella sonnei* | N/A | 53G-SP |  | (50) |
| *Shigella sonnei* | N/A | 53G-SP(pZA-P_SS_:*gfp*) |  | This study |
| *Shigella sonnei* | N/A | 53G-SP(pZA-P_SF2a_:*gfp*) |  | This study |
| *Shigella sonnei* | N/A | 53G-SP(pZA-P_SF6_:*gfp*) |  | This study |
| *Shigella sonnei* | N/A | 53G-SP Δetk |  | This study |
| *Shigella sonnei* | N/A | 53G-SP Δ*etk*(pBAD-*etk*) |  | This study |
| *Shigella sonnei* | N/A | 53G-SP Δ*etk*(pBAD) |  | This study |
| *Shigella sonnei* | N/A | 53G-SP ΔO-Ag |  | (50) |
| *Shigella sonnei* | N/A | 53G -pINV |  | (76) |
| *Shigella sonnei* | N/A | CS14 |  | (51) |
| *Shigella sonnei* | N/A | 100296 | Gambia | (9, 26) |
| *Shigella sonnei* | N/A | 204219 | Mali | (9, 26) |
| *Shigella sonnei* | N/A | 303522 | Mozambique | (9, 26) |
| *Shigella sonnei* | N/A | 401266 | Kenya | (9, 26) |
| *Shigella sonnei* | N/A | 503236 | India | (9, 26) |
| *Shigella sonnei* | N/A | 600202 | Bangladesh | (9, 26) |
| *Shigella sonnei* | N/A | 702021 | Pakistan | (9, 26) |
| *Shigella boydii* | 2 | 103335 | Gambia | (9, 26) |
| *Shigella boydii* | 4 | 100464 | Gambia | (9, 26) |
| *Shigella boydii* | 4 | 100464(pZA-P_SS_:*gfp*) |  | This study |
| *Shigella boydii* | 4 | 100464(pZA-P_SF2a_:*gfp*) |  | This study |
| *Shigella boydii* | 4 | 100464(pZA-P_SF6_:*gfp*) |  | This study |
| *Shigella dysenteriae* | 3 | 100310 | Gambia | (9, 26) |
| *Shigella dysenteriae* | 12 | 402253 | Kenya | (9, 26) |
| *Shigella dysenteriae* | 12 | 402253(pZA-P_SS_:*gfp*) |  | This study |
| *Shigella dysenteriae* | 12 | 402253(pZA-P_SF2a_:*gfp*) |  | This study |
| *Shigella dysenteriae* | 12 | 402253(pZA-P_SF6_:*gfp*) |  | This study |
| *Shigella flexneri* | 2a | 2457T |  | (77) |
| *Shigella flexneri* | 2a | 2457T(pBAD-*etk*) |  | This study |
| *Shigella flexneri* | 2a | 2457T(pBAD) |  | This study |
| *Shigella flexneri* | 2a | 2457T(pZA-P_SS_:*gfp*) |  | This study |
| *Shigella flexneri* | 2a | 2457T(pZA-P_SF2a_:*gfp*) |  | This study |
| *Shigella flexneri* | 2a | 2457T(pZA-P_SF6_:*gfp*) |  | This study |
| *Shigella flexneri* | 2a | 100060 | Gambia | (9, 26) |
| *Shigella flexneri* | 2a | 100060(pBAD-*etk*) |  | This study |
| *Shigella flexneri* | 2a | 500482 | India | (9, 26) |
| *Shigella flexneri* | 2a | 500482(pBAD-*etk*) |  | This study |
| *Shigella flexneri* | 3a | J17B |  |  |
| *Shigella flexneri* | 3a | J17B(pZA-P_SS_:*gfp*) |  | This study |
| *Shigella flexneri* | 3a | J17B(pZA-P_SF2a_:*gfp*) |  | This study |
| *Shigella flexneri* | 3a | J17B(pZA-P_SF6_:*gfp*) |  | This study |
| *Shigella flexneri* | 4a | 402059 | Kenya | (9, 26) |
| *Shigella flexneri* | 4a | 702013 | Pakistan | (9, 26) |
| *Shigella flexneri* | 5a | M90T |  | (78) |
| *Shigella flexneri* | 5a | M90T(pZA-P_SS_:*gfp*) |  | This study |
| *Shigella flexneri* | 5a | M90T(pZA-P_SF2a_:*gfp*) |  | This study |
| *Shigella flexneri* | 5a | M90T(pZA-P_SF6_:*gfp*) |  | This study |
| *Shigella flexneri* | 6 | CCH060 |  |  |
| *Shigella flexneri* | 6 | CCH060(pZA-P_SS_:*gfp*) |  | This study |
| *Shigella flexneri* | 6 | CCH060(pZA-P_SF2a_:*gfp*) |  | This study |
| *Shigella flexneri* | 6 | CCH060(pZA-P_SF6_:*gfp*) |  | This study |
| *Shigella flexneri* | 6 | CCH060Δ*etk* |  | This study |
| *Shigella flexneri* | 6 | CCH060Δ*etk*(pBAD-*etk*) |  | This study |
| *Shigella flexneri* | 7a | 600471 | Bangladesh | (9, 26) |
| *Shigella flexneri* | 7a | 601016 | Bangladesh | (9, 26) |
| *Escherichia coli* | N/A | DH5a(pZA) |  | This study |
| *Escherichia coli* | N/A | DH5a(pZA-P_SS_:*gfp*) |  | This study |
| *Escherichia coli* | N/A | DH5a(pZA-P_SF2a_:*gfp*) |  | This study |
| *Escherichia coli* | N/A | DH5a(pZA-P_SF6_:*gfp*) |  | This study |

**REFERENCES:**

76. Sayeed S, Brendler T, Davis M, Reaves L, Austin S. 2005. Surprising dependence on postsegregational killing of host cells for maintenance of the large virulence plasmid of *Shigella* *flexneri*. J Bacteriol 187:2768–2773. https://doi.org/10.1128/JB.187.8.2768-2773.2005

77. Formal SB, Dammin GJ, Labrec EH, Schneider H. 1958. Experimental *Shigella* infections: characteristics of a fatal infection produced in guinea pigs. J Bacteriol 75:604–610. https://doi.org/10.1128/jb.75.5.604-610.1958

78. Sansonetti PJ, Kopecko DJ, Formal SB. 1982. Involvement of a plasmid in the invasive ability of *Shigella* *flexneri*. Infect Immun 35:852–860. https://doi.org/10.1128/iai.35.3.852-860.1982

| **Supplemental Table 2: Primers and plasmids used in this study** | | | |
| --- | --- | --- | --- |
| **Primer*/Plasmid** | **Source** | **Description** | **Sequence (5′→3′)** |
| 1F |  | Lambda red linear fragment in 53G SP | TGAGATCGATCTGCTTCGTCTGGTCGGCGAGTTATGGGATCACCGTAAGTTTATTATCAGCGTGACCGCGTTATTCACGCTGATCGCT**GTGTGTAGGCTGGAGCTGCTTC** |
| 1R |  | Lambda red linear fragment in 53G SP | ATCATATCGCTCAGGCCGCTGAGAATGGCGTTGCCCTGTTTTTGCTCAACCTGGACCAGAGTATCTGCCTGATAAATTGGTGTGCTTA**CATATGAATATCCTCCTTA** |
| 2F |  | Lambda red linear fragment in CCH060 | TACGCCACCAGGCAGCACTCAGGAAAATGAGATCGATCTGCTTCGTCTGGTCGGCGAGTTATGGGATCACCGTAAGTTTATTATCAGCGTT**GTGTAGGCTGGAGCTGCTTC** |
| 2R |  | Lambda red linear fragment in CCH060 | CGATCACCGCTGCCAGAGTTGAACTGACAAACGTTTTACCACTGTCTGGCGTCGCACCGGTGATCATCAGAATGTTATTCTCCGTCTCCATC**ATAGCATATGAATATCCTCCTTA** |
| 3F |  | Complementation into pBAD **BamHI** | GTCA**GGATCC**ATGACAACTAAAAATATGAATACGCCACCA |
| 3R |  | Complementation into pBAD **AvrII** | CACT**CCTAGG**TTACTCTTTCTCGGAGTAACTATAACCG |
| ymcD_F_NheI |  |  | GACA**GCTAGC**ACAGCCAGTTATAGTACCACTTGAAAC |
| G4C_GFP_F |  | binds to GFP with tail that is homologous to *gfc* 3’-native promoters | GATGAATAAAGGAAAAGTTATGAGTAAAGGAGAAGAACTTTTCACTGGAGTTG |
| G4C_GFP_R |  | Binds to *gfc* 3’-native promoter with tail that is homologous to GFP | AAGTTCTTCTCCTTTACTCATAACTTTTCCTTTATTCATCGCATGGACAA |
| GFP_R_AvrII |  |  | GACT**CCTAGG**TAGGCGGGCTAGCTCATTATTTG |
| pZA_001 |  | Screening PCR for positive mutants | AGTCACTCAATCCTCTTCACAAC |
| pZA_002 |  | Screening PCR for positive mutants | TCACTTCCCTGTTAAGTATCTCC |
| pKD46 | (63) |  |  |
| pKD4 | (63) |  |  |
| pCP20 | (63) |  |  |
| pBAD |  |  |  |
| pBAD-*etk* | This study |  |  |
| pGEN206-GFP | (65, 66) |  |  |
| pZA112-trc | (64) |  |  |
| pZA(-)*trc* | This study |  |  |
| pZA-PSS:*gfp* | This study |  |  |
| pZA-PSF2a:*gfp* | This study |  |  |
| pZA-PSF6:*gfp* | This study |  |  |

*Primers used for PCR and sequencing are based on GenBank HE616528.1 and CP099865.1 and were generated by Sigma.

**Supplemental Figure 1: Expression of Etk in clinical isolates of *Sf*2a strains does not reduce invasion efficiency in HT-29 cells.** Human HT-29 cell monolayers were infected with WT *Ss* 53G, *Sf*2a strains 2457T, 100060, and 500482, and *Sf*2a strains containing pBAD-*etk* for 90 minutes, treated with gentamicin for 30 minutes, washed, and lysed to determine intracellular bacterial counts expressed as % invasion (CFU recovery). Bacteria were grown in TSB to mid log and *etk* expression was induced in pBAD-*etk* with 0.03% arabinose for 15 minutes. Data presented is a representative assay from 2 independent experiments. Each dot represents data collected from an individual monolayer. Asterisks indicate statistically significant differences determined using one-way ANOVA with Šídák’s multiple-comparison test. *,*P*<0.05; **,*P*<0.01.

**Supplemental Figure 2: The *gfc* native promoter for *Ss* 53G and *Sf*6 CCHO60 both express GFP while the promoter for *Sf*2a does not.** All *E.coli* and *Shigella* strains were plated on minimal media and GFP fluorescence was visualized via an ultraviolet transilluminator. DH5α (A), *Sf*2a 2457T (B), *Sf*3a J17B (C), *Sf*5a M90T (D), *Sf*6 CCH060 (E), and *Ss* 53G (F). No plasmid control (1), pZA-P_SS_:*gfp* (2), pZA-P_SF2a_:*gfp* (3), or pZA-P_SF6_:*gfp* (4).
